# Machine Learning for Evaluating the Cytotoxicity of Mixtures of Nano-TiO_2_ and Heavy Metals: QSAR Model Apply Random Forest Algorithm after Clustering Analysis

**DOI:** 10.3390/molecules27186125

**Published:** 2022-09-19

**Authors:** Leqi Sang, Yunlin Wang, Cheng Zong, Pengfei Wang, Huazhong Zhang, Dan Guo, Beilei Yuan, Yong Pan

**Affiliations:** 1 College of Safety Science and Engineering, Nanjing Tech University, Nanjing 211816, China; 2 Department of Emergency Medicine, The First Affiliated Hospital of Nanjing Medical University, Nanjing 210006, China; 3 Department of Preventive Health Branch, The Affiliated Jiangning Hospital of Nanjing Medical University, Nanjing 211100, China

**Keywords:** QSAR, AdaBoost, RF, cluster analysis, mixture, cytotoxicity, quantum mechanics

## Abstract

With the development and application of nanomaterials, their impact on the environment and organisms has attracted attention. As a common nanomaterial, nano-titanium dioxide (nano-TiO_2_) has adsorption properties to heavy metals in the environment. Quantitative structure-activity relationship (QSAR) is often used to predict the cytotoxicity of a single substance. However, there is little research on the toxicity of interaction between nanomaterials and other substances. In this study, we exposed human renal cortex proximal tubule epithelial (HK-2) cells to mixtures of eight heavy metals with nano-TiO_2_, measured absorbance values by CCK-8, and calculated cell viability. PLS and two ensemble learning algorithms are used to build multiple QSAR models for data sets, and the test set R^2^ is increased from 0.38 to 0.78 and 0.85, and RMSE is decreased from 0.18 to 0.12 and 0.10. After selecting the better random forest algorithm, the K-means clustering algorithm is used to continue to optimize the model, increasing the test set R^2^ to 0.95 and decreasing the RMSE to 0.08 and 0.06. As a reliable machine algorithm, random forest can be used to predict the toxicity of the mixture of nano-metal oxides and heavy metals. The cluster analysis can effectively improve the stability and predictability of the model, and provide a new idea for the prediction of cytotoxicity model in the future.

## 1. Introduction

Nanotechnology is continually developing, and nanomaterials are gradually applied in various fields. With these developments, an interest has arisen to determine the possible risks in the production and use of nanomaterials. Because of nanomaterials’ small particle size and the increasing probability of contact with humans, nanoparticles can easily enter the human body and cause adverse effects [1]. Therefore, cytotoxicity research of nanomaterials is essential. Nano-metal oxides are widely used in industrial and daily fields [2], and the market is developing rapidly. As a common nano-metal oxide, nano-titanium dioxide (nano-TiO_2_) is continuously evaluated. Many studies have recorded its cytotoxic characteristics, such as weathering resistance [3], photocatalytic performance [4], and composite materials [5]. In addition to the well-known characteristic functions of nano-TiO_2_, including high specific surface area, stability, anti-corrosion, and photocatalysis [6], it can also have sterilization [7], UV protection [8], and self-cleaning functions [9]. Therefore, it has been mass-produced and widely used. The prospects for development are promising.

Due to its properties, such as small particle size, high specific surface area, and high active surface [10], nanoparticles are highly susceptible to adsorption with other substances to form mixtures. This results in a shift in their effects on the environment and organisms [11]. Some nano-metal oxide particles have obvious toxicity, and the induction of biological cytotoxicity will inhibit the growth of organisms and cause their death [12]. Considering the above factors, the cytotoxicity of mixtures of nano-TiO_2_ with other substances have been studied to evaluate the biological toxicity of nano-TiO_2_ with other pollutants in the environment. With the current industrialization processes, heavy metal compounds have been widely used in production and application, and heavy metal pollution has begun to appear in some areas [13], causing serious environmental harm. Unlike organic pollutants, these pollutants are not easily decomposed, leading to long-term accumulation and ultimately causing heavily biological hazards [14]. Given that nanomaterials can adsorb heavy metals [15], joint organismal toxicity should be established.

**Figure 1:**
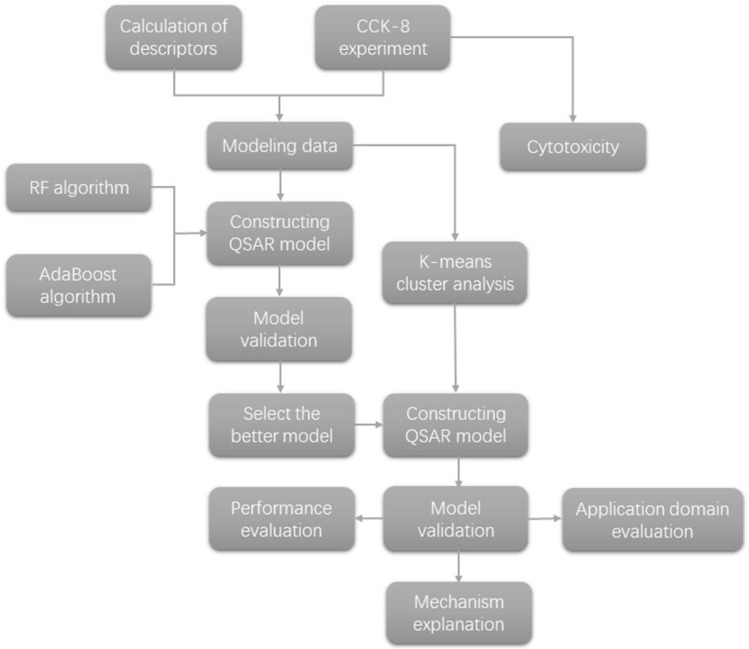
The flowchart of the work.

In recent years, Quantitative Structure-Activity Relationship (QSAR) models have been applied to investigate the cytotoxicity of nanomaterials [16,17,18,19]. The QSAR model is aimed at exploring the relationship between activity and structure. Materials with a similar chemical structure likely produce similar toxicity through comparable mechanisms. Therefore, Read-across [20] is used in nanotoxicity assessment, that is, the prediction of unknown data of substances with similar structure to known data substances. With the development of machine learning, machine learning algorithms can build mathematical models and make predictions based on training data [21]. Periodic table descriptors [22,23,24] are widely used in the study of nano-QSAR, but it is difficult to describe mixtures. Quantum mechanical descriptors [25] can accurately describe the electronic structure and reactivity of molecules, and are suitable for the toxicity prediction model of complex compounds or mixtures. Common algorithms include multiple linear regression (MLR) [26], partial least squares (PLS) [27], principal component regression (PCR) [28], and so on. There is a need for a complete QSAR model that is more applicable to various research predictions. Nano-QSAR is used to efficiently study nanoparticles and determine the correlation between nano-structure and biological activity. Metal oxide nanoparticles are the most thoroughly studied in nano-QSAR [29]. In addition to selecting optimal descriptors [30], ensemble learning algorithms have also been used in QSAR research. Ensemble learning algorithms are divided into Bagging and Boosting schools. There is a dependency relationship between classifiers in the Boosting school, which must be serial, such as AdaBoost [31]. There is no dependency between classifiers in Bagging school, and they can be parallel, such as random forest. The random forest algorithm [32,33,34,35,36] integrates multiple trees through ensemble learning. Its basic unit is the decision tree, and its essence belongs to a large branch of machine learning—the Ensemble Learning Algorithms. Compared to other algorithms, the random forest algorithm performs better in the accuracy of prediction results [37], but it is rarely used to predict cytotoxicity. The weights of decision trees in the random forest algorithm are the same, but the accuracy of decision trees is high or low. The AdaBoost algorithm is an optimization for this situation. Different weak models are trained for the same training set and combined to form a stronger model. Similar to the random forest algorithm, the AdaBoost algorithm is easier to adjust parameters than traditional algorithm. Both of them are algorithms based on the idea of ensemble learning, which can avoid certain over-fitting problems and make the model have better generalization ability and higher accuracy. Each tree in the random forest is independent, which is easier to be parallel and has high training efficiency. However, AdaBoost can’t train in parallel because every weak model depends on the previous weak model. Therefore, when PLS algorithm is not ideal, we use random forest and AdaBoost ensemble learning algorithms to model respectively, and finally choose the better random forest algorithm to establish QSAR model of cytotoxicity. Even though the mixtures of heavy metals with nano-TiO_2_ can be categorized, non-negligible differences were found. For substances with large descriptor gaps, removing them from the model would inevitably decrease the model’s accuracy. After eliminating this situation, we tried to classify the selected mixtures [38]. The K-means algorithm [39] is an unsupervised clustering algorithm, widely used because of its simple implementation and proper clustering, and can be used for the QSAR modeling process. In this study, the cytotoxicity data of proximal tubule epithelial cells of human renal cortex were measured in laboratory, and QSAR model was constructed by using the random forest algorithm after cluster analysis, and the validation indexes were compared, which provided some reference for further study of cytotoxicity model of metal oxide and heavy metal mixture. A neat flowchart of the work is shown in Figure 1.

## 2. Results and Discussion

### 2.1. Experimental Results

The results of the CCK-8 assay on that the toxicity of 25 μmol/L nano-TiO_2_ was low, the cell survival rate was close to 1, and the toxicity increased when mixed with heavy metals. HK-2 cells were exposed to eight mixtures for 24 h showed different degrees of apoptosis, and the cell survival rate decreased with the increase of the concentration of heavy metal compounds. The mixture of ZnCl_2_ and 25 μmol/L nano-TiO_2_ decreased the survival rate of HK-2 cells significantly when the concentration of ZnCl_2_ increased from 240 μmol/L to 270 μmol/L. Compared with the other six heavy metal compounds, Pb (NO_3_)_2_ and SbCl_3_ showed higher cytotoxicity in mixtures with 25 μmol/L nano-TiO_2_. The cell survival rate of the eight mixtures is shown in Supplementary Material (Table S1), and the serial number corresponds to the serial number of the concentration in Table 1.

### 2.2. QSAR Model Calculation Results 

The partial least squares (PLS) [40] is a numerical algorithm which is widely used in QSAR modeling in recent years. It was first used in the establishment of this model. R^2^ > 0.6 is the first step for us to judge the quality of the model, but in PLS model, no matter how the data set is divided, the R^2^ of the test set in the optimal model is only 0.38, far below the standard of 0.6, and the predicted value of the model is not credible. RMSE (test) is 0.18. R^2^ and RMSE of the training set are 0.08 and 0.27 respectively. The comparison chart between the predicted value and the observed value of PLS model is shown in Supplementary Material (Figure S1), and the application domain is shown in Supplementary Material (Figure S2). Then, we use two typical machine ensemble learning methods, random forest and AdaBoost, to model 72 sample data.

All descriptors were put into the model, and the appropriate descriptors were screened out to fit the model. The selection of descriptors is actually consistent with the modeling method. “AdaBoostRegressor” and “RandomForestRegressor” in sklearn datasets are quoted respectively, and set the parameters “n_estimators” and “random_state” to 1~100 for fitting. The random state and the number of decision trees when R^2^ is the maximum are determined by grid search. Through modeling, the importance of each descriptor is calculated, and the descriptor whose “feature_importances_” is greater than 0.1 is selected to re-fit the model. The importance of the optimized descriptors of AdaBoost model and RF model is shown in Table 2, from which it can be seen that absolute hardness and adsorption energy have shown high importance in these two models. 

**Figure 2:**
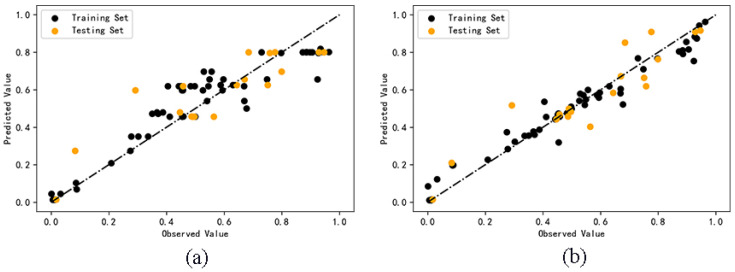
Comparison of observed and predicted values of AdaBoost (**a**) and RF (**b**) models.

The predicted values of cell survival rate are shown in Supplementary Material (Table S1). The comparison between the predicted values and the observed values of the two models is shown in Figure 2. According to the degree of dispersion of scattered points in the figure, the accuracy of the predicted value can be seen more intuitively. The dispersion degree of prediction results of random forest model is smaller than that of AdaBoost model, which indicates that the model based on the random forest algorithm is more accurate than that of the AdaBoost algorithm. 

In order to explore the influence of cluster analysis on QSAR modeling, the random forest algorithm was selected to continue the study. The K-means clustering algorithm is used to classify all data into Category 1 and Category 2. Category 1 included CdCl_2_, ZnCl_2_, MnCl_2_, and CoCl_2_. Category 2 included CuSO_4_, NiCl_2_, Pb (NO_3_)_2_, and SbCl_3_. Model A is a random forest model built by Category 1. Model B is a random forest model built by Category 2. Set the parameter cycle from 1 to 100, determine the parameters of “n_estimators” and “random_state” when R^2^ is the maximum, and select the descriptors whose “feature_importances_” is greater than 0.1 to re-model. Model A and B were created to model the Category 1 and Category 2 datasets separately to observe the influence of different categories on the predicted activity results. After screening and re-fitting, the importance of the optimization descriptors of Model A and B is shown in Table 2. Combined with the four models, absolute hardness and adsorption energy are two descriptors whose importance is always high. In addition, the importance of Lowest orbital energy in model A can’t be ignored.

**Figure 3:**
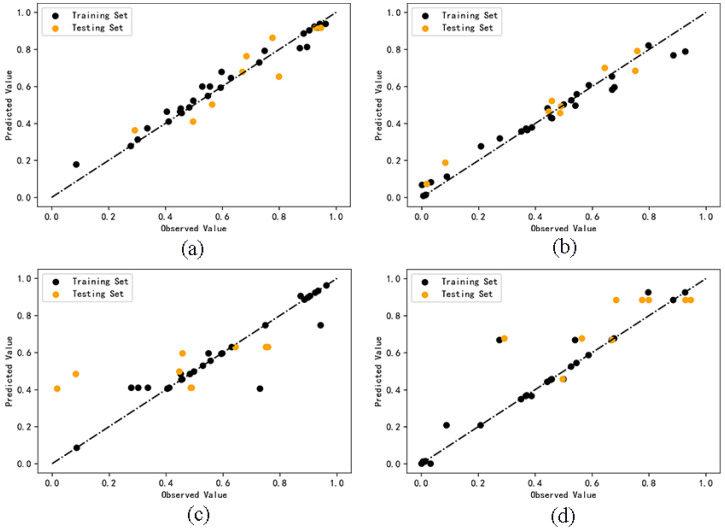
Comparison between observed and predicted values of Model A (**a**), B (**b**), C (**c**), D (**d**).

The comparison between the predicted values and the observed values of the two cluster analysis models is shown in Figure 3. The cluster analysis model based on random forest algorithm shows a low degree of dispersion and a more accurate prediction ability.

For Model C and D, we used cross-prediction methods between the training and the test set. Model C used random forest algorithm to model the training set of Category 1 and predict the test set of Category 2. Model D used random forest algorithm to model the training set of Category 2 and predict the test set of Category 1. Set the parameter cycle from 1 to 100, determine the parameters of “n_estimators” and “random_state” when R^2^ is the maximum, and build the model. The results show that the R^2^ of Model C test set is 0.31, and that the R^2^ of Model D test set is 0.35, both of which are far lower than 0.6, indicating that the reliability of the predicted values is low and the generalization ability of the model is insufficient. The result of the model is too poor, so it is not meaningful to screen descriptors to optimize the model. The comparison between the predicted values and the observed values of the two cross models is shown in Figure 3. The dispersion degree of Model C and D is much higher than that Model A and B. Obviously, the model fitted by Category 1 samples is not suitable for the test set of Category 2. The model fitted by Category 2 samples is not suitable for the test set of Category 1. It shows that it is significant to build the model separately after cluster analysis, which can theoretically improve the accuracy of the model and will be further explained by the validation results of the model.

### 2.3. Model Validation Results

The QSAR model must be validated to provide a reasonable explanation for data analysis. The larger the squared correlation coefficient (R^2^), the smaller the root mean square error (RMSE), and the larger the correlation coefficient ($Q_{LOO}^{2}$) of Leave-one-out (LOO) cross-validation usually means that the model has better prediction ability and robustness. RMSE is more reliable than R^2^ [41]. The Y randomization correlation coefficient ($R_{yrand}^{2}$) and Y randomization correlation coefficient ($Q_{yrand}^{2})$ both had low values. The above belongs to the internal validation of the model. The larger $Q_{F1}^{2}$,$Q_{F2}^{2}$,$Q_{F3}^{2}$ and the concordance correlation coefficient (CCC), the better the generalization ability of the model. The specific model validation criteria were as follows:$\;Q_{LOO}^{2}$ > 0.5 [42],$\;R_{yrand}^{2}$ < 0.5, $Q_{yrand}^{2}$ < 0.5 [43], RMSE < 0.2, R^2^ > 0.6, $Q_{F1}^{2}$ > 0.5 [44], $Q_{F2}^{2}$ > 0.5 [45], $Q_{F3}^{2}$ > 0.5 [46], CCC > 0.85 [47]. The number of samples in training and test sets, the optimal parameters N estimators and Random state of AdaBoost, Random Forest, A, B, C and D models and their corresponding validation results are shown in Table 3. Comparing the validation parameters of random forest algorithm and AdaBoost algorithm, we find that for the same sample set, all the validation parameters of random forest algorithm are far better than AdaBoost algorithm. The $Q_{F3}^{2}$ value of the model based on AdaBoost algorithm is lower than the evaluation standard, which shows that the generalization ability of this model is insufficient. Because AdaBoost algorithm is much more sensitive to noise than random forest algorithm, the model built by AdaBoost algorithm is inferior to random forest algorithm in generalization ability, robustness and prediction ability. The validation parameters of Model A and B meet the model evaluation standard. Most of the validation parameters of models C and D don’t meet the model evaluation criteria. By comparing the validation parameters of RF, A and B models in Table 3, it can be clearly seen that all the validation parameters in Model A and B are better than those of the random forest model. which shows that the clustering analysis model has better prediction ability, robustness and generalization ability in this case. 

### 2.4. Application domain analysis

**Figure 4:**
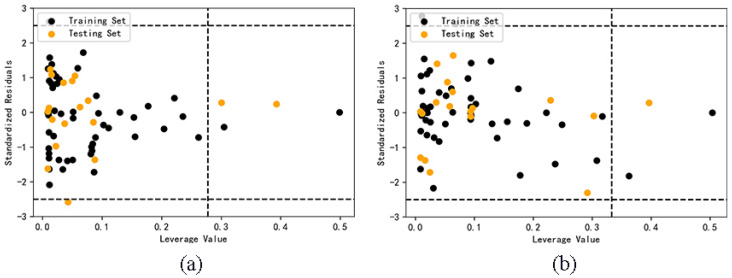
Application domain of AdaBoost (**a**) and RF (**b**) models.

Only the samples in the application domain (AD) can explain the reliability of the predicted value. The critical leverage value of AdaBoost model and RF model before classification are 0.2778 and 0.3333. It can be seen from Figure 4 that four samples of AdaBoost model are outside the application domain, and three samples of RF model are outside the application domain. The result is limited by the definition of application domain. The decrease of descriptors and the increase of the number of samples can both lead to the decrease of the critical value of leverage value, thus narrowing the scope of application, and it is easy for some samples to exceed the application domain. This shows that if the model with relatively large data set is optimized by selecting descriptors, the reliability of the model may be reduced.

**Figure 5:**
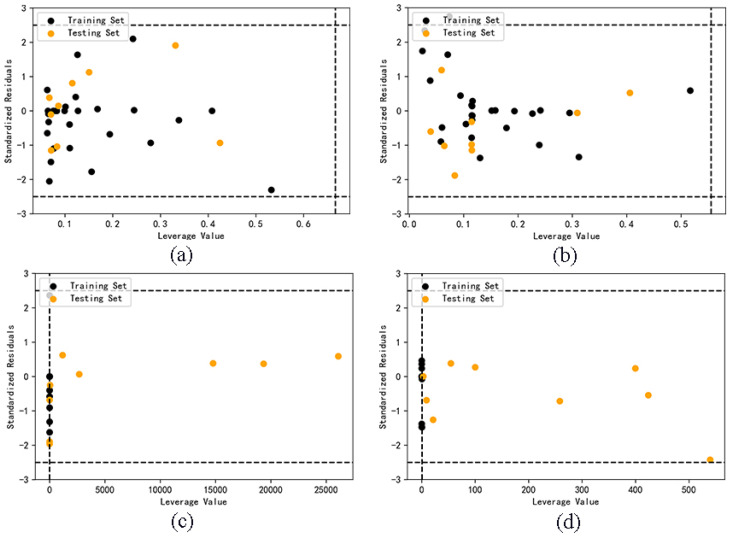
Application domain of Model A (**a**), B (**b**), C (**c**), D (**d**).

The critical leverage values of Model A and B are 0.6667 and 0.5556 respectively. It can be seen from Figure 5 that all samples of Model A and B are in the application domain, indicating that the prediction results of these two models are all reliable. However, almost all the test set samples in Model C and D do not belong to their corresponding application domain. Compared with the first two models, the applicability and accuracy of Model C and D are obviously reduced, and the classification model is not suitable for cross prediction of test set samples.

### 2.5. Research Results of the Toxicity Mechanisms

Comparing the screening results of descriptors from different samples, we found that the three characteristics of lowest orbital energy, absolute hardness and adsorption energy have important influence on the survival rate of HK-2 cells, and absolute hardness and adsorption energy are of high importance in all models. To a certain extent, the lowest orbital energy reflects the oxidation ability of substances as oxidants. The smaller the lowest orbital energy, the more favorable it is for electrons to be filled. Externally, the stronger the oxidation ability, the direct influence on the concentration of reactive oxygen species (ROS) in cells. Absolute hardness represents the difference between ionization potential and electron affinity, which essentially reflects the difficulty of gaining or losing electrons, that is, this feature also represents the oxidation of materials. Adsorption can indicate the degree of amount of energy required for different particles to be adsorbed together. The larger the adsorption energy, the easier it is to cause unstable atoms to separate from the material surface. Through the above mechanism analysis, we think that the mixture of nano-materials and heavy metals can damage HK-2 cells mainly by promoting the generation of free radicals.

We measured ROS of some mixtures to explore the cytotoxicity mechanisms of mixtures of nano-TiO_2_ and heavy metals [48]. The results showed that this type of mixture increases intracellular hydroxyl radicals (OH^−^) when it leads to cell apoptosis, which is an oxidative stress reaction [49]. With the increase of the concentration of heavy metals in the mixture, the concentration of ROS generally increased (Figure S3), which indicated that the mixture of nanomaterials and heavy metals induced cell death by increasing the concentration of ROS in HK-2 cells.

## 3. Materials and Methods

### 3.1. Cell Experiments

HK-2 cells were cultured in Hyclone DMEM medium supplemented with 10% fetal bovine serum (FBS) and 100 units/ml penicillin/streptomycin and incubated at 37 ℃ and 5% CO_2_. Nano-TiO_2_ and heavy metal powders were mixed in the prepared medium. In addition to setting the concentration of nano-TiO_2_ at 25 μmol/L, nine concentrations of heavy metals were set, as shown in Table 1.

HK-2 cells were cultured in 96-well plates at 37 ℃ and 5% CO_2_ for 24 h. After reaching 70% confluence, the prepared mixture of nano-TiO_2_ and heavy metals was added. A blank control group with cells without venom was set up. After 24 h of culture at 37 ℃ and 5% CO_2_, the supernatant was removed, and 100 μL CCK-8(Cell Counting Kit-8) liquid was added to each well. After 30 min, each well’s absorbance was measured by using a microplate reader with a wavelength of 450 nm. The Equation (1) used for calculating the cell viability is as follows:

(1) $$S=\frac{A_{\exp}-A_{\mathrm{blank}}}{A_{\mathrm{control}}-A_{\mathrm{blank}}}$$

S is the cell survival rate, $A_{\exp}$ is the absorbance value of the experimental group, $A_{\mathrm{control}}$ is the absorbance value of the control group, and $A_{\mathrm{blank}}$ is the absorbance value of the blank control group.

### 3.2. Research on the QSAR Model

#### 3.2.1. Selection and Calculation of Descriptors

Based on the mixture’s characteristics, we used quantum mechanical descriptors [50,51] to establish the QSAR model of cytotoxicity of nano-TiO_2_ mixtures with heavy metals. These descriptors included highest orbital energy, lowest orbital energy, ionization potential, electron affinity, absolute electronegativity, absolute hardness, molecular energy, and adsorption energy [52]. Based on density functional theory (DFT), B3LYP functional and LANL2DZ basis set are used in Gaussian [53] to optimize the structure and calculate highest orbital energy, lowest orbital energy and molecular energy. After obtaining the quantum mechanical (QM) characteristics of each material, the remaining descriptors are deduced by using specific formulas. Specific formulas are referred to in Supplementary Material (Table S2). 

Generally, there are two mathematical models to calculate the toxicity mechanism of mixtures: concentration addition (CA) and independent action (IA) [54]. Except the adsorption of nano-TiO_2_ on heavy metals, there is almost no other interaction and reaction between the two components, so other descriptors of the mixture were calculated by CA model. The calculation Equation (2) used was:

(2) $$D_{mix}=\overset{n}{\underset{i=1}{\sum }}D_{i}x_{i}$$

$D_{mix}$ is the descriptor of the mixture, $D_{i}$ is the descriptor of component *i*, and $x_{i\;}$ is the molar concentration of component *i*.

All final descriptor data are referred to in Supplementary Material (Table S1).

In the actual model construction process, we found that not all molecular descriptors are suitable for building models, and only the descriptors that have great influence on the results are reserved. Descriptors whose importance is less than 0.1 are usually considered as unimportant features, which are screened out because they have little influence on the prediction results. Some specific related descriptors may come from the same structural factors [55], and finally only one was selected to be included in the model. 

The methods of selecting descriptors include heuristic method (HM), genetic algorithm (GA), random forests (RF), etc. In the random forest algorithm, feature importance refers to the contribution of each feature to each tree in a random forest, and compares the contribution between features after taking the average value. Gini index [56] or out-of-bag (OOB) error [57] can usually be used as an evaluation index. In this paper, we used the output variable “feature_importances_” in sklearn datasets, and set the R^2^ of training set and test set to be the maximum to terminate the filtering. That is, Gini importance is used to sort the features, and sklearn normalizes all Gini importance in the form of sum to obtain the final output parameter of “feature_importances_”.

#### 3.2.2. Classification of Mixture Types

For the eight heavy metals included in this study, we use Python software to classify the mixture types. We randomly selected two as the initial clustering centers. We calculated the Euclidean distance [58] from each point to the center of the cluster through descriptors and delimited the nearest one to the cluster center. Every time a sample was assigned, the cluster center was recalculated according to the existing objects in the cluster, and finally, the heavy metal sample set was divided into two clusters. According to the mixture descriptor, the mixture was divided into two categories. Category 1 included CdCl_2_, ZnCl_2_, MnCl_2_, and CoCl_2_. Category 2 included CuSO_4_, NiCl_2_, Pb (NO_3_)_2_, and SbCl_3_. 

#### 3.2.3. Data Set Division

An essential step in QSAR research is dividing the data set into a training set and a test set. We performed this division according to the 3: 1 by random sampling (RS) method. In this study, eight heavy metals were researched. Nine concentrations of each heavy metal were selected and mixed with 25 μmol/L nano-TiO_2_. We obtained data from 72 samples. Therefore, 54 samples were randomly selected for the development model’s training set, and the remaining 18 were placed into the test set to evaluate the model’s predictability. For classified samples, 27 samples were randomly selected as the training set of the development model, and the remaining 9 samples were put into the test set to evaluate the predictability of the model.

#### 3.2.4. Algorithm Application

In the random forest algorithm, it is necessary to classify the input samples and enter that sample into each tree for classification. Firstly, it will randomly sample the data sample set for N times and get a subset of the training set as the new training set (this sampling method is called the bootstrap sample method) [59]. Secondly, in the new training set, K attributes will be randomly extracted from the attribute set of characteristic variables as the attribute subset, and an optimal attribute will be selected from the attribute subset for node splitting. Finally, based on generating M decision trees, the classification result is decided by voting of M decision trees to form a random forest.

The AdaBoost algorithm gets a series of weak classifiers through repeated learning and then combines them to get a strong classifier. Firstly, the same weights are given to N training sample data. Secondly, when constructing the next training set, the weight of the training sample points accurately classified by the weak classifier will decrease, otherwise, the weight will increase. After the weight is updated, a new weak classifier will be added for iteration every round. Finally, increase the weight of weak classifiers with low classification error rate, and reduce the weight of weak classifiers with high classification error rate, so that weak classifiers with low classification error rate will play a decisive role, otherwise, they will be combined into the final strong classifier.

#### 3.2.5. Model Validation

The internal validation of the QSAR model mainly validate s the fitting ability and robustness of the model; the external validation mainly aims at the model’s prediction ability. In addition to the commonly used the squared correlation coefficient (R^2^) [60] and root mean square error (RMSE) [61] for internal validation, we also used the leave-one-out (LOO) cross-validation method [62] to evaluate the internal prediction ability of the model by calculating the correlation coefficient ($Q_{LOO}^{2}$) between the predicted value (ŷ_i_) and the observed value (y_i_) to reduce the probability of model overfitting. The y randomization correlation coefficient ($R_{yrand}^{2}$) and the y randomization Q^2^ ($Q_{yrand}^{2}$) values generated by the Y randomization validation [43] validate the model’s robustness to avoid accidental correlation. With the external validation of the model, we validated the model’s generalization ability by the index $Q_{F1}^{2}$, $Q_{F2}^{2}$, $Q_{F3}^{2}$ and the concordance correlation coefficient (CCC). The above evaluation indexes are all calculated by specific formulas. Specific formulas for model validation indexes are referred to in Supplementary Material (Table S3).

#### 3.2.6. Application Domain of the Model

The application domain (AD) is a spatial region defined by the training set samples’ descriptors and the studied properties. Only the compounds in the space can be considered reliable, and the samples beyond the application domain cannot guarantee the accuracy of the results. We used Williams diagram to analyze the application domain of the QSAR model, the ordinate represents the standardized residual, the abscissa represents the sample leverage value. The calculation method of leverage value is shown in Equation (3):

(3) $$h_{i}=x_{i}{\left(X^{T}X\right)}^{-1}x_{i}^{T}\;\;\;\;i=1,2\ldots \ldots ,n$$

$x_{i}$ represents the sample descriptor, and X represents the sample set composed of all the training set descriptors. 

We take ±2.5 standardized residuals as the critical values. When the standardized residuals of the predicted values are greater than the critical values or the leverage value $h_{i}$ of the samples to be tested is greater than the critical values h*, the prediction quality is low. The calculation method of lever critical value is shown in Equation (4):

(4) $$h*=\frac{3\left(p+1\right)}{n}$$

p represents the number of descriptors, and n represents the number of samples in the training set.

## 4. Conclusions

We determined the cytotoxicity of mixtures of eight heavy metal compounds and nano-TiO_2_ on HK-2 cells and explored the toxicity mechanism. We also used partial least squares (PLS) to establish a QSAR model. We showed that the test set R^2^ and RMSE of this model are 0.38 and 0.18 respectively, suggesting that the modeling effect is poor. The partial least squares (PLS) is the most useful data analysis method used by most scholars to determine cytotoxicity, but this method has some limitations. We then introduced the random forest algorithm and AdaBoost algorithm for QSAR modeling. These two machine ensemble learning methods allow to evaluate the cell survival rate, and improve the test set R^2^ to 0.78 and 0.85, and RMSE is decreased to 0.12 and 0.10. Among them, the random forest model has better prediction ability, robustness and generalization ability, and the feasibility of the model was proven. Therefore, when the standard regression method (such as PLS) performs poorly in the model, we can try to introduce ensemble learning methods, such as random forest algorithm. In addition, the K-means clustering algorithm improves the R^2^ to 0.95 and decreases RMSE to 0.08 and 0.06 after classification, which shows the advantages of the model after clustering analysis.

## Figures and Tables

**Table 1:** The concentration of heavy ions.

Serial Number	CdCl_2_ (μmol/L)	ZnCl_2_ (μmol/L)	CuSO_4_ (μmol/L)	NiCl_2_ (μmol/L)	Pb(NO_3_)_2_ (μmol/L)	MnCl_2_ (μmol/L)	SbCl_3_ (μmol/L)	CoCl_2_ (μmol/L)
1	10	60	30	100	100	100	5	10
2	20	90	60	200	200	200	10	20
3	30	120	90	300	300	300	15	30
4	40	150	120	400	400	400	20	40
5	50	180	150	500	500	500	25	50
6	60	210	180	600	600	600	30	60
7	70	240	210	700	700	700	35	70
8	80	270	240	800	800	800	40	80
9	90	300	270	900	900	900	45	90

**Table 2:** The importance weight of feature in RF and AdaBoost.

Descriptor	AdaBoost	RF	Model A	Model B
Highest orbital energy				
Lowest orbital energy		0.15	0.39	
Ionization potentials		0.10		
Electron affinity	0.14		0.07	
Absolute electronegativity			0.16	0.25
Absolute hardness	0.25	0.20	0.22	0.22
Molecular energy	0.11	0.14		0.31
Adsorption energy	0.49	0.40	0.16	0.21

RF refers to random forest algorithm. Models A and B are based on random forest algorithm.

**Table 3:** The internal and external validation results of models.

Model Parameters	AdaBoost	RF	Model A	Model B	Model C	Model D
Training set samples	54	54	27	27	27	27
Test set samples	18	18	9	9	9	9
N estimators	8	4	4	9	1	1
Random state	93	35	95	83	19	79
R^2^ (train)	0.86	0.95	0.97	0.97	0.88	0.90
R^2^ (test)	0.78	0.85	0.85	0.95	0.31	0.35
RMSE (train)	0.10	0.06	0.04	0.05	0.08	0.09
RMSE (test)	0.12	0.10	0.08	0.06	0.20	0.16
$Q_{\mathrm{Loo}}^{2}$	0.69	0.70	0.73	0.81	−0.06	0.64
$R_{yrand}^{2}$	−0.20	−0.44	−0.45	−0.47	−0.86	−0.79
$Q_{\mathrm{yrand}}^{2}$	−0.25	−0.45	−0.49	−0.50	−1.01	−1.03
$Q_{F1}^{2}$	0.79	0.86	0.87	0.95	0.50	0.79
$Q_{F2}^{2}$	0.78	0.85	0.85	0.95	0.31	0.35
$Q_{F3}^{2}$	0.37	0.57	0.61	0.85	−0.51	0.36
CCC	0.87	0.92	0.93	0.97	0.43	0.62

RF refers to random forest algorithm. Models A, B, C and D are based on random forest algorithm.

## Supplementary Materials

The following supporting information can be downloaded at: https://www.mdpi.com/article/10.3390/molecules27186125/s1. Model details (including descriptor data, observed and predicted values of cell survival rate, data set division) (Table S1) (EXCEL); Specific formulas for deducing descriptors (Table S2); Specific formulas for model validation (Table S3); Comparison of observed and predicted values of PLS (Figure S1); Application domain of PLS (Figure S2); ROS value (Figure S3) (PDF).
