# Supplementary material for: Machine Learning for Evaluating the Cytotoxicity of Mixtures of Nano-TiO2 and Heavy Metals: QSAR Model Apply Random Forest Algorithm after Clustering Analysis
Source: Molecules. 2022 Sep 19;27(18):6125. doi: 10.3390/molecules27186125 (PMC9500633; doi:10.3390/molecules27186125)
Supplement: Supplementary file 1 [file molecules-27-06125-s001.zip › molecules-1862861-supplementary/Supplementary Material.pdf]

Supplementary material

Refer to Table S2 for specific formulas for deducing descriptors.

Refer to Table S3 for specific formulas for model verification.

Refer to Figure S1 for the comparison of observed and predicted values of PLS.

Refer to Figure S2 for the application domain of PLS.

Refer to Figure S3 for the ROS value.

Table S2 Specific formulas for deducing descriptors

Descriptor	Formula	Description
Ionization potential	I=$E_c^{N-1} - E_0^N$	$E_c^{N-1}$is the energy after losing one electron
Electron affinity	A=$E_0^N - E_A^{N+1}$	$E_0^N$is the basal state energy(neutral)<br>$E_A^{N+1}$is the energy after gaining one electron
Absolute electronegativity	u=$\frac{I+A}{2}$	u is absolute electronegativity<br>I is ionization potential
Absolute hardness	η=$\frac{I-A}{2}$	A is electron affinity<br>η is absolute hardness
Adsorption energy	ΔE_{ads}=$E_{tot} - \sum_{i=1}^{n} E_i$	E_{tot} is the total energy of mixture<br>$\sum_{i=1}^{n} E_i$ is the sum of energy of all materials

Table S3 Specific formulas for model verification

Index	Formula	Description
R^2	$R^2 = 1 - \frac{\sum_{i=1}^{n}(y_i - \hat{y}_i)^2}{\sum_{i=1}^{n}(y_i - \bar{y}_i)^2}$	
RMSE	RMSE=$\sqrt{\frac{\sum_{i=1}^{n}(y_i-\hat{y}_i)^2}{n}}$	y_i is the observed value<br>$\hat{y}_i$ is the predicted value<br>$\bar{y}_i$ is the average value of the observed values<br>n is the number of samples
Q_{LOO}^2	Q_{LOO}^2=1-$\frac{\sum_{i=1}^{n}(y_i-\hat{y}_i)^2}{\sum_{i=1}^{n}(y_i-\bar{y}_i)^2}$	$\bar{y}_{TR}$ is the average value of the observed values in the training set
Q_{F1}^2	Q_{F1}^2=1-$\frac{\sum_{i=1}^{n_{EXT}}(\hat{y}_i-y_i)^2}{\sum_{i=1}^{n_{ext}}(y_i-\bar{y}_{TR})^2}$	$\bar{y}_{EXT}$ is the average value of the observed values in the test set
Q_{F2}^2	Q_{F2}^2=1-$\frac{\sum_{i=1}^{n_{EXT}}(\hat{y}_i-y_i)^2}{\sum_{i=1}^{n_{EXT}}(y_i-\bar{y}_{EXT})^2}$	n_{TR} is he number of training set samples<br>n_{EXT} is he number of test set samples
Q_{F3}^2	Q_{F3}^2=1-$\frac{\sum_{i=1}^{n_{EXT}}(\hat{y}_i-y_i)^2/n_{EXT}}{\sum_{i=1}^{n_{ext}}(y_i-\bar{y}_{TR})^2/n_{TR}}$	

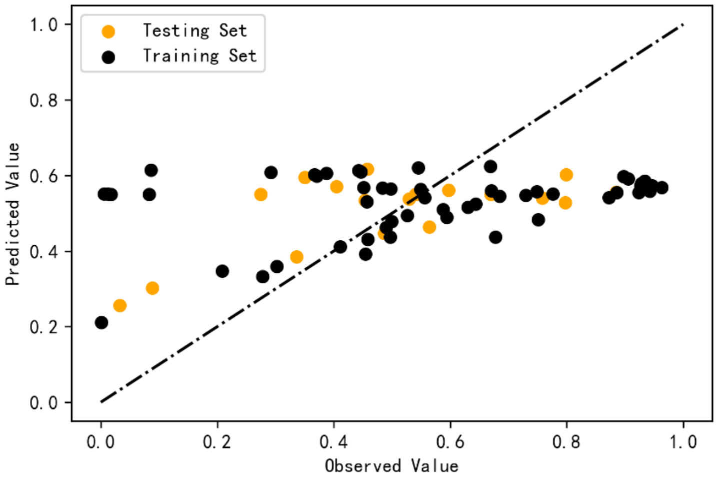

Figure S1 Comparison of observed and predicted values of PLS

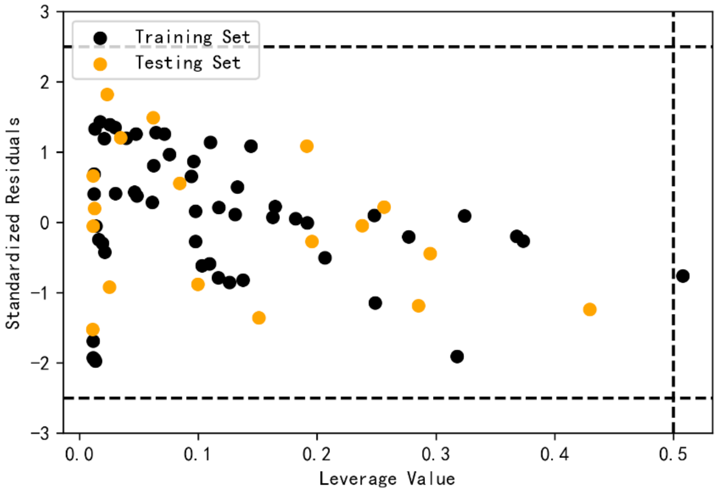

Figure S2 Application domain of PLS

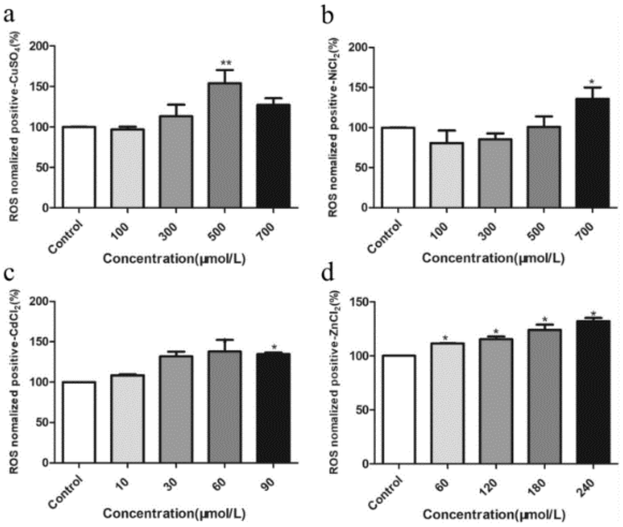

Figure S3 ROS value
